# A feasibility trial of delayed resection for brain metastases following pre-operative stereotactic radiosurgery

**DOI:** 10.1007/s11060-025-05081-2

**Published:** 2025-05-26

**Authors:** Christina Schröder, Neda Haghighi, Claire Phillips, Cristian Udovicich, Michelle P. Li, Katharine Drummond, James Dimou, Andrew S. Davidson, Joseph Sia

**Affiliations:** 1 Department of Radiation Oncology, Peter MacCallum Cancer Centre, Melbourne, VIC 3000 Australia; 2 Department of Radiation Oncology, Inselspital, Bern University Hospital, University of Bern, Bern, Switzerland; 3 Department of Neurosurgery, The Royal Melbourne Hospital, Parkville, Australia; 4 Department of Surgery, The University of Melbourne, Melbourne, Australia; 5 Sir Peter MacCallum Department of Oncology, The University of Melbourne, Melbourne, Australia; 6 GenesisCare Radiation Oncology, Melbourne, Australia; 7 Personalised Oncology Division, Walter and Eliza Hall Institute of Medical Research, Melbourne, Australia

**Keywords:** Brain metastases, Stereotactic radiosurgery, Radiation therapy, Neurosurgery, Neuro-oncology, Pre-operative SRS

## Abstract

**Purpose:**

Pre-operative stereotactic radiosurgery (SRS) for brain metastases (BrM), an emerging alternative to post-operative SRS, is typically performed 1–2 days before resection. However, a longer period of the irradiated tumour in situ may confer anti-tumour immunological benefits. We conducted the first clinical trial to evaluate the feasibility of planned delayed resection after pre-operative SRS.

**Methods:**

In this single-arm trial, patients with suspected BrM suitable for pre-operative SRS and surgery were eligible. The primary endpoint was feasibility of resection 7–21 days after SRS, with a pre-defined feasibility threshold of 66% receiving this. Secondary endpoints included 6-month adverse events (AE) and local control (LC) rates. Tumour volume change was assessed from SRS- and neurosurgery-planning MRI’s.

**Result:**

78 patients were screened and the target accrual of 15 patients was met. Common reasons for pre-operative SRS ineligibility were lack of existing cancer diagnosis (44%) and tumour size/peri-tumoural oedema (18%). Two patients declined resection after SRS. The median SRS-to-surgery interval was 8 days (range 0–15). Nine tumours in 8 patients (56%) received delayed resection. Reasons for earlier resection were predominantly non-medical. There were no Grade > 2 AE. The 6-month BrM LC was 100%. At a median follow-up of 13.8 months, the only BrM local failure after SRS and resection occurred with a 0-day SRS-to-surgery interval. No histopathological diagnosis issues were encountered with delayed resection. An increased SRS-to-surgery interval correlated with greater tumour shrinkage.

**Conclusions:**

The pre-defined feasibility threshold for delayed resection was not met, but more than half of patients received delayed resection without safety concerns.

**Trial registration number:**

ACTRN12622001372774 (Registered 26/10/2022).

**Supplementary Information:**

The online version contains supplementary material available at 10.1007/s11060-025-05081-2.

## Introduction

Stereotactic radiosurgery (SRS) is a highly precise form of ablative radiation therapy for intracranial tumours. It is a key treatment for patients with brain metastases (BrM), either as a standalone approach or in combination with surgical resection [1]. Post-operative SRS to the resection cavity has become an important tool for achieving improved local disease control following surgical resection of BrM [2]. However, this requires a generous radiation volume encompassing the cavity, surgical tract, and surrounding meningeal margins, as well as managing uncertainties in target delineation due to post-operative changes and cavity dynamics [3, 4]. Pre-operative SRS is an emerging alternative that addresses these issues. Non-randomised studies suggest it may reduce risks of regional leptomeningeal disease (LMD) and radionecrosis due to pre-operative tumour cell sterilisation and a smaller irradiated volume, while maintaining comparable or even superior local control, owing to a more radiosensitive (less hypoxic) and potentially immunologically active in situ tumour microenvironment [5–12].

However, factors that influence the interval between pre-operative SRS and resection are not well characterised. In published studies, BrM resection has typically occurred within 2 days of SRS [5–11], though whether this timing is driven by clinical or logistical factors is unclear. Currently, no guidelines define the ideal resection timing after pre-operative SRS. Where medically appropriate, an argument to extend the interval between pre-operative SRS and resection is to allow sufficient time for SRS-induced tumour-directed immune responses to develop within the irradiated tumour, while in situ. Studies in murine and human tumours suggest DNA damage repair and cell death processes predominate the immediate post irradiation phase, but anti-tumour adaptive immune responses require 1–2 weeks to form [13–15]. A pre-clinical study found that tumour resection 1 day after irradiation did not generate tumour-directed immunological memory, but delaying resection until day 7 did [16]. In parallel, recent investigations on SRS-treated patient BrM samples observed that T cell infiltrates decreased immediately following SRS but significantly rebounded after 6 days [17, 18]. Suggesting a therapeutic implication of these findings, clinical studies suggest definitive SRS for in situ BrM and immune checkpoint blockade (ICB) could act synergistically to improve local and systemic disease control [19, 20].

To date, only 4 single-arm prospective feasibility and Phase 2 studies of pre-operative SRS for BrM have reported outcomes [5–8]. Here, we report a novel clinical trial examining the feasibility of planned delayed resection (7–21 days) following pre-operative SRS for BrM. This timeframe was selected because SRS-induced adaptive immune responses are likely to have developed in the irradiated tumour during this window, which will be examined in a subsequent translational sub-study. We also describe the clinical, histopathological and radiological outcomes of pre-operative SRS in this prospective cohort, as well as trial factors that may help inform the design of similar future clinical trials on pre-operative SRS timing.

## Materials and methods

### Study design

This was a single-centre, single-arm trial at the Peter MacCallum Cancer Centre (PMCC) and Royal Melbourne Hospital, Australia (ACTRN12622001372774). All patients referred to our neurosurgery service and multi-disciplinary meetings for resection of suspected BrM based on radiological and clinical characteristics were screened for suitability to receive pre-operative SRS and enrolment into study. Inclusion criteria included age ≥ 18 years and medical fitness for surgery. Patients could have any number of BrM but must have ≥ 1 BrM considered for resection. Considerations for suitability for pre-operative SRS including mass effect and neurological deficits were made by the treating neurosurgeon and radiation oncologist. Exclusion criteria were presence of radiological diffuse LMD and pregnancy. In the initial trial protocol, eligibility was restricted to non-small cell lung cancer (NSCLC) and melanoma histology types and dexamethasone use of ≤ 4 mg/day. These were removed on protocol amendments four and eight months into study to improve accrual. Additional BrM not requiring surgery were managed as per standard of care. Dexamethasone use was at the discretion of the treating clinicians and did not necessarily reflect BrM-related symptoms.

Timing of resection following pre-operative SRS was decided jointly between the neurosurgical and radiation oncology teams after study enrolment, accounting for medical (such as need for urgent resection or rapid commencement of systemic therapy) and logistical (such as length of inpatient stay and theatre availability) factors. For patients enrolled on study, a delayed resection (7–21 days) was preferred where possible. All study participants provided written informed consent. This study was conducted with approval from the PMCC’s Human Research Ethics Committee.

### Study endpoints

The primary endpoint was the feasibility of a 7-21-day time window between pre-operative SRS and resection of the SRS-treated BrM, which was defined a priori to be met if 66% of patients underwent BrM resection in that timeframe. Secondary endpoints were adverse events, wound healing complication, local control (LC), distant brain control (DBC), LMD, radionecrosis, and overall survival (OS) rates at 6 months. LC, DBC and OS beyond this protocol-specified 6-month follow-up period were determined by retrospective review of medical records.

LC was evaluated on magnetic resonance imaging (MRI) of the brain and defined as the absence of emergence or progression (a 20% increase in diameter) of nodular contrast-enhancing lesions within the resection cavity. DBC was defined as the absence of progressive disease at intracranial sites that did not receive SRS, as determined by the RANO-BM criteria [21]. Development of LMD was assessed by the reporting neuroradiologist and categorised as either local (limited to around the surgical cavity) or diffuse. Radionecrosis was either diagnosed histologically, if resected, or radiologically based on MRI appearances and temporal characteristics in a multi-disciplinary setting [22, 23]. The date of first radiological diagnosis of radionecrosis may be made retrospectively, as an observation period of lesion stability or regression over time may be required. OS was defined as absence of death from any cause. Adverse events and wound healing complication were assessed at follow-up time points (refer below) and graded according to the Common Terminology Criteria for Adverse Events (CTCAE) version 5.0.

### Treatment and follow-up

Pre-operative SRS was delivered either using the Leksell Gamma Knife Icon or a Varian TrueBeam linear accelerator (LINAC). SRS dose-fractionation prescription was at the discretion of the treating radiation oncologist but is generally based on BrM size. To facilitate comparison across SRS dose-fractionation schedules, biologically equivalent doses were calculated using an α/β of 10 (BED_10_) [24]. Isotropic margins of 0 mm and 1 mm were used for Gamma Knife and LINAC treatments respectively to generate planning tumour volumes (PTV). The covering isodose prescription was variable for GK treatments, based on dosimetric parameters such as coverage and selectivity, and generally to 80–90% for LINAC treatments.

After SRS, patients were followed up at 1 week, 4 weeks, 3 months and 6 months following SRS. A detailed medical history, relevant physical examination and adverse event assessment were performed at each time point. Post-treatment surveillance MRI scans of the brain were performed at 3 and 6 months following pre-operative SRS in this trial, which included pre- and post-contrast T1 MPRAGE, T1 SPACE, T2, FLAIR, and DWI sequences. Peripheral blood samples were collected at baseline, 1 week, 4 weeks and 3 months, in addition to the resected tumour, for a translational sub-study.

Following pre-operative SRS, a repeat stereotactic MRI was generally performed for surgical planning, guided by the treating neurosurgeon. The SRS-planning (pre-SRS) and neurosurgery-planning (post-SRS, pre-resection) stereotactic MRI scans were imported into a radiation treatment planning system for contouring of SRS-treated tumours by the same investigator to assess volumetric change following SRS.

### Statistical analysis

A sample size of 15 patients was set as a pragmatic target. The primary endpoint was defined a priori to be met if 66% of patients underwent BrM resection 7–21 days following pre-operative SRS. This threshold was deemed the minimum required to suggest capacity for consistent uptake while accounting for unexpected variations. For the secondary endpoints of LC, DBC, LMD, radionecrosis and OS rates, the Kaplan-Meier method was used to estimate medians and 95% confidence intervals (CI), calculated from completion of pre-operative SRS. LC, LMD and radionecrosis rates were calculated at a per lesion level, while DBC and OS rates were calculated at a per treatment episode (as defined by a new SRS-planning MRI) and per patient levels, respectively. Patients who remained alive and progression-free at time of analysis were censored at date of last radiological follow-up. Volumetric change was correlated with time between SRS-planning and neurosurgery-planning MRI’s using linear regression. Statistical significance was set at a threshold of *p* < 0.05.

## Results

### Factors affecting suitability for pre-operative SRS

**Fig. 1:**
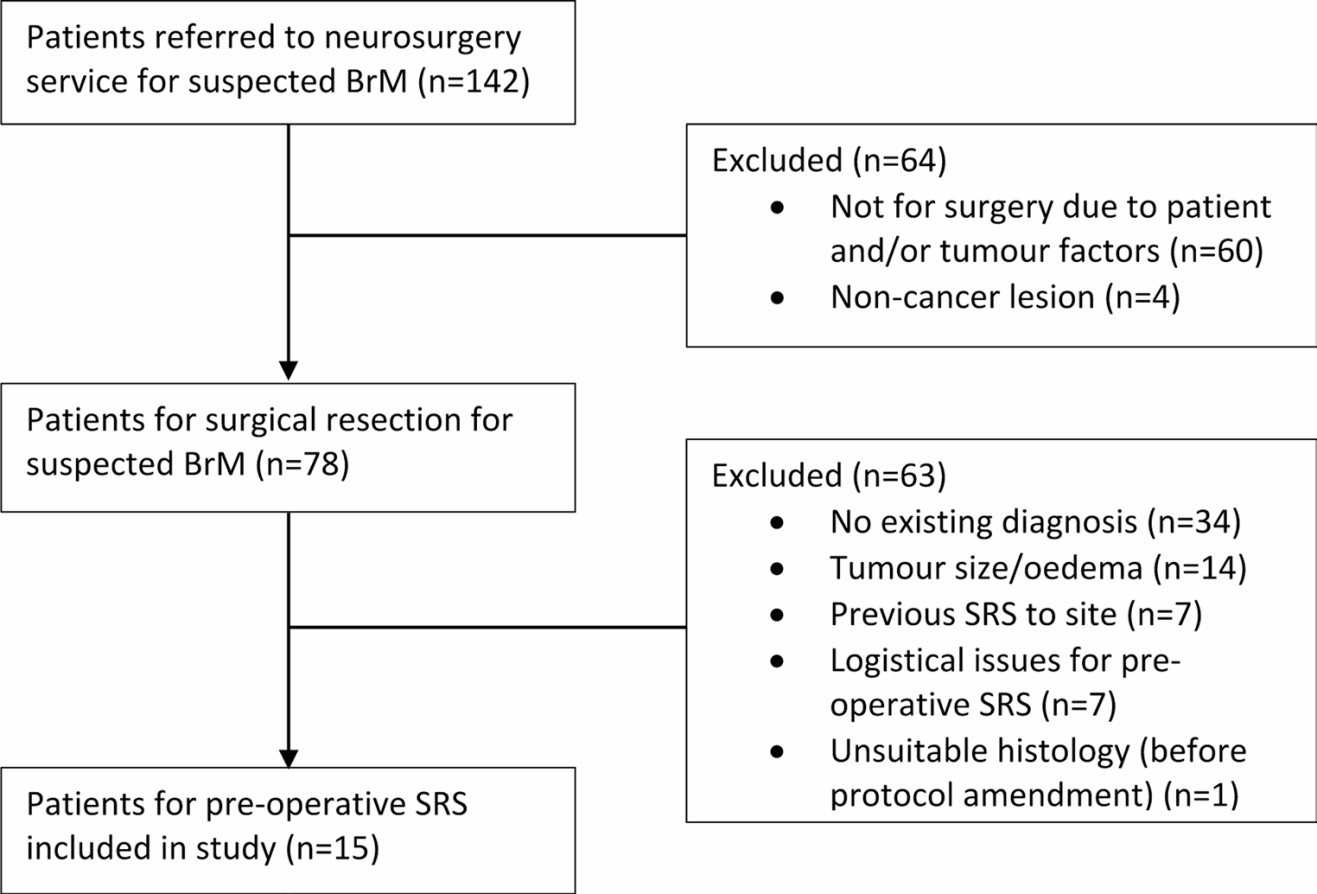
CONSORT diagram of trial

The study opened in January 2023 and completed accrual in August 2024. 78 patients for surgical resection of a suspected BrM were screened, of whom 15 patients were enrolled in this study (Fig. 1). The most common reasons for ineligibility were a lack of existing cancer diagnosis and/or need for histopathological confirmation (44%), as well as mass effect from the tumour and/or peri-tumoural oedema (18%).

### Patient and tumour characteristics

Patient and tumour characteristics are outlined in Table 1 and Supplementary Table 1. The median age was 53 years (range 26–76 years). Two patients declined surgery after completion of SRS. Another patient received pre-operative SRS and resection for 2 tumours over 2 separate episodes. Thus, 14 tumours in 13 patients were resected following pre-operative SRS.

**Table 1:** Patient and tumour characteristics

	*n* (%)
**Per patient** (*n* = 15)	
**Age**,** years** (median [range])	53 [29–76]
**Sex**	
Male	8 (47)
Female	7 (53)
**ECOG Performance Status**	
0	6 (40)
1	9 (60)
**Per episode / tumour** (*n* = 16)	
**Histology**	
Melanoma	5 (31)
Colorectal	3 (19)
Breast	3 (19)
NSCLC	3 (19)
Sarcoma	1 (6)
Astrocytoma	1 (6)
**Tumour diameter**,** mm** (median, [range])	25 [15–56]
**Brain region**	
Frontal	5 (31)
Parietal	5 (31)
Temporal	2 (13)
Cerebellar	4 (25)
**Systemic anti-cancer therapy**	
Ipilimumab/Nivolumab	3 (19)
Atezolizumab	1 (6)
Lorlatinib	1 (6)
None	11 (69)
**Dexamethasone use**	
0 mg	4 (25)
2 mg	2 (13)
4 mg	7 (44)
8 mg	3 (18)
**SRS dose schedule**	
24 Gy in 3 fractions (BED_10_ 43 Gy)	3 (19)
27 Gy in 3 fractions (BED_10_ 51 Gy)	5 (31)
25 Gy in 5 fractions (BED_10_ 38 Gy)	3 (19)
Other	5 (31)

BED_10_: biologically equivalent dose, assuming α/β = 10

The most common histology was melanoma (*n* = 5, 31%), followed by non-small cell lung (NSCLC), breast and colorectal cancers (*n* = 3, 19% each). One patient presented with a solitary contrast-enhancing cystic brain tumour (15 mm diameter) on a history of HER2-amplified breast cancer. This was presumed to be a BrM after multi-disciplinary review and received pre-operative SRS and resection. The initial histopathology revealed a WHO Grade 2 astrocytoma, IDH-mutant, but on further completion surgery to resect a small residual non-contrast enhancing tumour component, this returned to be WHO Grade 4 astrocytoma, IDH-mutant.

The median tumour diameter was 25 mm (range 15–56 mm) and the median SRS gross target volume (GTV) was 10.4 cc (range 3.0-53.3 cc). The most common SRS dose-fractionation schedules were 27 Gy in 3 fractions and 25 Gy in 5 fractions (*n* = 3, 19% each). Twelve tumours were treated on the GammaKnife and 4 on the LINAC. All patients completed the prescribed SRS treatments.

### Time from pre-operative SRS to resection

Nine tumours in 8 patients (56%) underwent resection 7–21 days after SRS, thus not meeting the pre-defined criterion for feasibility of delayed resection. Of the tumours that were resected, the median time from completion of SRS to resection was 8 days (range 0–15 days).

Reasons for earlier resection were logistical (*n* = 2, due to theatre and inpatient bed availabilities), non-medical preferences (*n* = 2, due to approaching social commitments), and medical (*n* = 1, to expedite systemic therapy due to significant extracranial disease) (details in Supplementary Table 1). Other than a small difference in dexamethasone dose at time of SRS, there were no significant differences in patient and tumour factors between those receiving delayed resection and early or no resection (Supplementary Table 2).

### Adverse events

There was minimal toxicity within 6 months following SRS (Table 2). Two patients developed Grade 2 headaches at 1- and 4-weeks following SRS; the former was assessed as likely related to SRS and treated with commencement of dexamethasone, while the latter was assessed as likely related to surgery (occurring over craniotomy site) and treated with tapentadol. Two patients reported Grade 2 fatigue at 1- and 4-weeks following SRS. There were no Grade ≥ 3 adverse events and no complications with wound healing.

**Table 2:** Incidence of adverse events within 6 months

	Grade 1	Grade 2	Grade 3	Grade 4
Headaches	1	2	0	0
Fatigue	1	2	0	0
Nausea	1	0	0	0
Alopecia	0	1	0	0

### Oncological outcomes

Thirteen patients (87%) completed protocol-specified 6 months follow-up. The 2 patients who dropped out of follow-up developed disease-related clinical deterioration and inability to attend appointments. The actuarial median follow-up time for the cohort was 13.8 months.

**Fig. 2:**
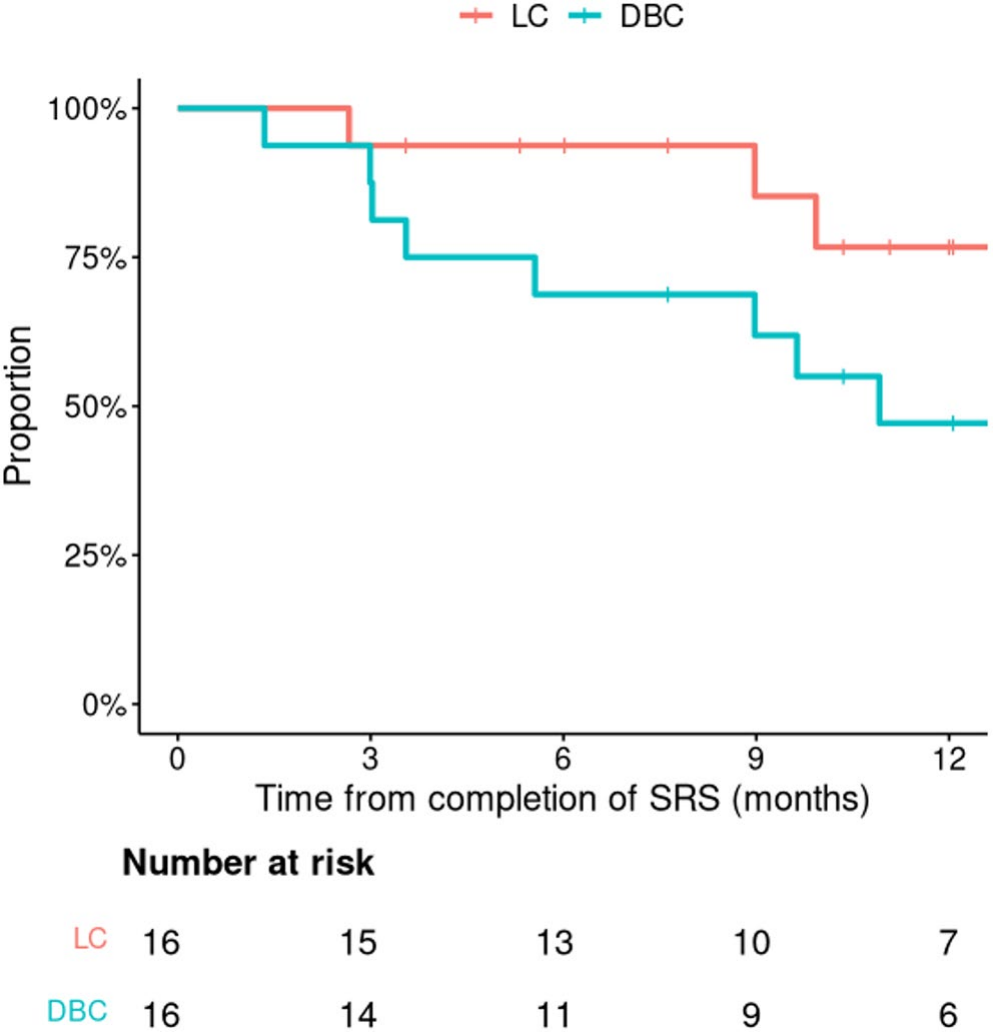
Local control (LC) and distant brain control (DBC) rates after SRS

There were 3 local failure events at time of analysis. The 6-month LC by intention-to-treat was 94% (95% CI 83–100%) (Fig. 2). However, the sole local failure within this period occurred in the case of unexpected glioma. For the BrM-only cohort, the 6- and 12-month LC rates were 100% and 82% (95% CI 62–100%). Interestingly, the only BrM local failure after pre-operative SRS and resection, at time of analysis, occurred in a patient who had a 0-day SRS-to-surgery interval (surgery on the same day as SRS). The other BrM local failure case occurred in a patient who received repeat SRS to the index lesion, 16 months following initial SRS, and subsequently refused surgical resection on trial.

There were 8 distant brain failure events at time of analysis. The 6-month DBC by intention-to-treat was 69% (95% CI 49–96%) (Fig. 2), and the median DBC duration was 10.9 months. The 6-month LMD relapse, radionecrosis, and death rates were 0%. At time of analysis, 3 deaths had occurred.

### Histopathological diagnosis after pre-operative SRS

In all 14 resected tumours (0–15 days following pre-operative SRS), a histopathological diagnosis could be made with no issues, even for those with delayed resection. One BrM had very few tumour cells present and was unsuitable for molecular testing, but this was likely due to the abundance of non-cellular material in a mucinous colorectal carcinoma histology.

### Tumour size dynamics after pre-operative SRS

**Fig. 3:**
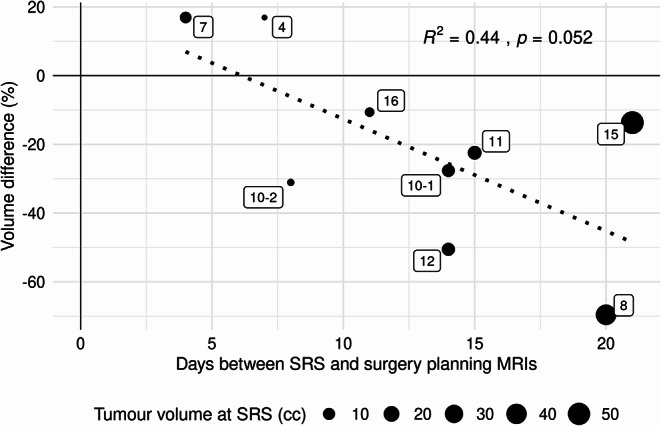
Correlation between tumour volume and time from SRS. Labels represent patient ID (corresponding to those in Supplementary Table 1)

Eight patients over 9 treatment episodes underwent both an MRI for pre-operative SRS and for neurosurgery planning. There was a near-significant trend for correlation between volume reduction and increasing time from completion of pre-operative SRS, though the model performance was mediocre (R^2^ = 0.44, *p* = 0.053, Fig. 3). In 6 patients, the tumour shrunk between scans by a median of 27.6% (range 10.6–69.6%). Interestingly, the magnitude of volume reduction was not dependent on the baseline tumour volume at time of SRS. Notably, the 2 tumours that had a radiological increase in volume (median 16.9%, range 16.9–16.9%) were the only tumours that were resected < 7 days from completion of pre-operative SRS.

## Discussion

Pre-operative SRS for BrM is an emerging alternative sequencing strategy to post-operative SRS, with Phase 3 randomised trials comparing the two approaches underway [25–27]. Given the potential impact of delayed resection timing on improved tumour-directed immunological responses to SRS, this study is the first to examine the feasibility of delayed resection following pre-operative SRS. Although the pre-defined feasibility criterion for delayed resection after pre-operative SRS was not met in this trial, more than half (56%) were able to undergo delayed resection, with an overall median time from SRS to surgery of 8 days. Notably, none of the cases for earlier resection were due to medical deterioration or SRS-induced oedema. There were no unexpected or serious adverse events and histopathological diagnosis was possible for all cases, corroborating the evolving literature that pre-operative SRS is safe [5–8]. Within this timeframe, a trend for correlation between radiological tumour shrinkage and delayed resection was seen. Intriguingly, the only BrM local failure following pre-operative SRS and resection occurred in a patient with a 0-day SRS-to-surgery interval (surgery on same day as SRS). Translational analysis of collected biospecimens to examine the immunological impact of SRS and timing to resection are underway.

In this study, two patients did not proceed with surgery following SRS, due to subsequent withdrawal of consent. This raises questions around selection of cases that categorically require bi-modality SRS and surgery (in contrast to SRS alone), and whether SRS dose-fractionation schedules should differ between definitive and pre-operative contexts. Radioresistant histology types and BrM causing significant mass effect are indications for surgical resection but often cases are less well-defined. In such cases, it may be that SRS alone, including hypofractionated SRS for larger BrM, is sufficient [28, 29]. Early studies of pre-operative SRS utilised single-fraction doses 10–20% below the maximum tolerated doses as determined in RTOG 90−05 [11, 30, 31]. Subsequent studies, including this current trial, have used definitive doses, including hypofractionated SRS, in the pre-operative setting with no signal for increased toxicity [5, 9, 10]. In fact, there is suggestion that hypofractionated SRS may offer better LC than single-fraction SRS in the pre-operative context [9], though this is not universally corroborated [10]. Altogether, we suggest there is no need to dose de-escalate in the pre-operative setting, ensuring the tumour has been well treated in case surgery ultimately does not proceed.

Our screening log highlights the challenges in patient accrual for a pre-operative SRS trial, namely the absence of a prior cancer diagnosis and need for histopathological confirmation, as well as tumour size and/or degree of peri-lesional oedema. A common concern with preoperative SRS is its potential impact on histopathological accuracy due to altered cellular morphology and reduced tumour cell viability post-irradiation [32, 33]. However, these changes accumulate over time and may not be significant in the short-to-intermediate period after irradiation. In this study, clear diagnoses were achieved for all tumour samples, including those from delayed resection (up to 15 days post-SRS). The only sample unsuitable for additional molecular testing had a low tumour cell count likely due to its intrinsic histology (mucinous colorectal carcinoma subtype) rather than effects of SRS. Although these findings alleviate concerns about pre-operative SRS compromising histopathological diagnoses, there remains a general risk that a SRS-treated tumour may have an unexpected histology [34]. In one case from this study, despite a strong pre-test probability of BrM, the SRS-treated tumour turned out to be a WHO Grade 4 astrocytoma, IDH-mutant. This patient subsequently completed full-dose adjuvant RT (60 Gy) with concurrent temozolomide without complications to date. However, this risk of unexpected histology is not unique to pre-operative SRS, but applies to all RT strategies when tissue diagnosis is not possible, such as curative-intent radiation therapy for presumed lung cancers where biopsy is deemed high-risk [35].

Secondly, tumour size and/or the degree of surrounding oedema are critical factors dictating the urgency for resection and thus suitability for pre-operative SRS, especially if the turnaround time for SRS (including treatment planning and quality assurance) is extended. Rather than specifying strict eligibility criteria for pre-operative SRS, our study left this decision to the clinical judgement of the treating neurosurgery and radiation oncology teams, which could have subjected accrual to varying comfort levels between clinicians. Other published and ongoing prospective trials have specific exclusion criteria such as a tumour size cut-off, midline shift, and signs of increased intracranial pressure to mitigate this potential subjectivity [5, 6]. This may be an important consideration, given BrM that require surgery are almost by default larger and/or have more peri-lesional oedema than those that do not.

This study has several limitations. First, the sample size was small with 15 patients (16 tumours). As discussed, we did not mandate specific tumour-related criteria for pre-operative SRS, which may have introduced variability in patient selection. Additionally, since the primary endpoint was the feasibility of delayed resection, the protocol-specified follow-up period was short for assessing secondary tumour control and radionecrosis endpoints. Despite these limitations, this study provides important insights in the pre-operative SRS scene as prospective data remain scarce, particularly regarding factors influencing adoption of the pre-operative SRS strategy and timing of resection following SRS. Further translational analysis from this study is awaited to determine the optimal timing of resection from an immunological perspective, particularly for the pre-operative SRS context when the tumour is in situ, as opposed to when the tumour bulk has been resected.

## Conclusion

As clinical and translational data around pre-operative SRS develop, the timing of resection following pre-operative SRS becomes increasingly important when considering biological windows of opportunity for leverage with systemic treatments such as immune-based therapies. Delayed resection appears to be safe with no early resection required for medical deterioration or SRS-related oedema. However, adaptations in hospital systems and clinician preferences are required for consistent adoption.

## Electronic supplementary material

Below is the link to the electronic supplementary material.

Supplementary Material 1

## Data Availability

No datasets were generated or analysed during the current study.
