# Supplementary material for: A feasibility trial of delayed resection for brain metastases following pre-operative stereotactic radiosurgery
Source: J Neurooncol. 2025 May 26;174(3):599–607. doi: 10.1007/s11060-025-05081-2 (PMC12263798; doi:10.1007/s11060-025-05081-2)
Supplement: Supplementary file 1 — Supplementary Material 1 [file 11060_2025_5081_MOESM1_ESM.docx]

| **Pt ID** | **Age** | **Sex** | **ECOG** | **Histology** | **BrM size (mm)** | **Brain region** | **Dex (mg/day)** | **SRS dose schedule** | **Days from SRS to surgery** | **Reason for shorter interval to surgery** |
| --- | --- | --- | --- | --- | --- | --- | --- | --- | --- | --- |
| 1 | 71 | M | 1 | Melanoma | 34 | Frontal | 2 | 27Gy/3fx | 12 |  |
| 2 | 76 | M | 0 | Melanoma | 20 | Frontal | 4 | 27Gy/3fx | NA | Patient refused surgery after SRS |
| 3 | 49 | F | 1 | NSCLC | 23 | Parietal | 4 | 24Gy/3fx | NA | Patient refused surgery after SRS |
| 4 | 49 | F | 1 | Astrocytoma | 15 | Frontal | 2 | 20Gy/1fx | 8 |  |
| 5 | 63 | M | 1 | CRC | 28 | Frontal | 4 | 27.5Gy/5fx | 10 |  |
| 6 | 59 | M | 1 | NSCLC | 18 | Cerebellar | 0 | 18Gy/1fx | 0 | Theatre time availability |
| 7 | 53 | F | 1 | NSCLC | 24 | Cerebellar | 8 | 27Gy/3fx | 0 | Bed availability – patient lived in a rural area and could not feasibly be discharged and return for surgery |
| 8 | 26 | M | 0 | Sarcoma | 51 | Parietal | 4 | 30Gy/5fx | 15 |  |
| 9 | 42 | M | 0 | Melanoma | 24 | Cerebellar | 4 | 24Gy/3fx | 2 | Surgeon preference - upcoming public holiday |
| 10 − 1 | 29 | M | 0 | CRC | 31 | Temporal | 4 | 27Gy/3fx | 15 |  |
| 11 | 50 | F | 0 | Breast | 25 | Cerebellar | 0 | 27Gy/3fx | 13 |  |
| 12 | 68 | M | 1 | Melanoma | 32 | Parietal | 0 | 25Gy/5fx | 11 |  |
| 10 − 2 | 29 | M | 0 | CRC | 21 | Temporal | 0 | 20Gy/1fx | 8 |  |
| 14 | 50 | F | 0 | Melanoma | 40 | Parietal | 8 | 25Gy/5fx | 3 | To expedite start of immunotherapy due to extracranial disease burden |
| 15 | 70 | F | 1 | Breast | 56 | Parietal | 4 | 25Gy/5fx | 7 |  |
| 16 | 56 | F | 1 | TNBC | 22 | Frontal | 8 | 24Gy/3fx | 3 | Patient preference - upcoming wedding |

*ECOG: Eastern Cooperative Oncology Group performance status; NSCLC: non-small cell lung cancer; CRC: colorectal cancer; Dex: dexamethasone; fx: fraction; NA: not applicable; Note patient #10 had two tumours resected over two episodes*

**Supplementary Table 1: Individual patient and tumour details**

| **Characteristic** | **Delayed** **resection**  (n = 9)*^a^* | **Early or no resection**  (n = 7)*^a^* | **p-value***^b^* |
| --- | --- | --- | --- |
| **Age (years)** | 50 [29, 68] | 53 [49, 59] | 0.71 |
| **Sex** |  |  | 0.66 |
| Female | 3 (33%) | 4 (57%) |  |
| Male | 6 (67%) | 3 (43%) |  |
| **ECOG performance status** |  |  | 1.00 |
| 0 | 4 (44%) | 3 (43%) |  |
| 1 | 5 (56%) | 4 (57%) |  |
| **Dexamethasone dose at time of SRS (mg/day)** | 2 [0, 4] | 4 [4, 8] | 0.044 |
| **BrM size (mm)** | 31 [25, 34] | 23 [20, 24] | 0.125 |
| **Brain region** |  |  | 0.362 |
| Cerebellar | 1 (11%) | 3 (43%) |  |
| Frontal | 3 (33%) | 2 (29%) |  |
| Parietal | 3 (33%) | 2 (29%) |  |
| Temporal | 2 (22%) | 0 (0%) |  |

*^a^Median [interquartile range]; n (%)*

*^b^Wilcoxon rank sum test; Fisher's exact test*

*ECOG: Eastern Cooperative Oncology Group*

**Supplementary Table 2: Patient and tumour factors by resection timing**
